# Objective Determination of Optimal Number of Spectral-Domain Optical Coherence Tomographic Images of Retina to Average

**DOI:** 10.1371/journal.pone.0110550

**Published:** 2014-10-22

**Authors:** Makoto Shirasawa, Taiji Sakamoto, Hiroto Terasaki, Takehiro Yamashita, Eisuke Uchino, Shozo Sonoda

**Affiliations:** Department of Ophthalmology, Kagoshima University Graduate School of Medical and Dental Sciences, Kagoshima, Japan; Univ Rochester Medical Ctr, United States of America

## Abstract

**Purpose:**

To determine by objective methods the minimum number of spectral-domain optical coherence tomographic (SD-OCT) images to average to obtain the clearest retinal image.

**Methods:**

SD-OCT Images were obtained from 9 healthy eyes and also from a phantom eye model. The SD-OCT images were obtained by averaging 1, 5, 20, 60, and 100 B-scan images. The reflectivity (mean gray value) of the different retinal layers was evaluated in these images. The image quality was evaluated by the size of the standard deviations (SDs) and the contrast-to-noise ratios (CNRs). A phantom eye model made by TiO_2_ silicone plates was also examined.

**Results:**

The SDs decreased significantly when the number of images averaged increased from 1 to 5 and also from 5 to 20 (*P*<0.05, post hoc Tukey's honestly significant difference tests). The SD of the automatic real time averaging of 1 (ART = 1) and ART = 5 were significantly larger than the SD of ART = 100 (*P*<0.05). The SDs of all other averaged numbers were not significantly larger than that of ART = 100. The CNR increased with an increase in the number of images averaged, and there was a significant increase between ART = 1 to 5 and between ART = 5 to 20 (*P*<0.05). No significant differences in the CNR was observed between ART = 5, ART = 20 and ART = 60. Similar results were obtained with the phantom eye model.

**Conclusions:**

Although the image quality of the SD-OCT images of the retina improved with an increase in the number of images averaged, it does not improve significantly by averaging more than 20 images.

## Introduction

Spectral-domain optical coherence tomographic (SD-OCT) instruments have higher scanning speeds, greater axial resolution, and higher sensitivity than time-domain OCT instruments, and these improvements have improved the clarity of the morphology of the retina and choroid [1], [2]. The major problem in obtaining clear SD-OCT images is speckle noise which significantly reduces the axial and transverse resolution of OCT instruments. To reduce speckle noise, multiple B-scan images are averaged [3]–[8]. In spite of the substantially faster rate of image acquisition by SD-OCT instruments, it still takes several seconds to obtain 100 overlapping images with the current SD-OCT instruments and even longer to acquire the images needed to construct a 3D structure of the eye.

A near infrared laser is commonly used as a light source in commercial OCT instruments, and the ability of near infrared light to damage the neurosensory retina and underlying structures is well known [9]. The American National Standards Institute standards for the safety of the retina to electromagnetic radiations considers the wavelength, exposure duration, and the number of exposures on the same spot of the retina [10]. Lasers are known to damage the retina [9], [11], but their safety has not been fully determined especially for repeated measurements. In addition, considering the need for frequent examinations to follow the natural course of some diseases or the effects of therapy, each session of measurements should be as short as possible. OCT examinations are also performed on young children who have limited attention spans and have difficulty maintaining steady fixation [12]. For these patients, the shortest measurement time is also important.

To counter these problems, multiple OCT images are recorded and averaged because it is known that the quality of OCT images improves with an increase in the number of images averaged [3], [4]. However, the increase in the number of images averaged will increase the exposure of the retina to the infrared illumination. Also, the increase in the number of images averaged will increase the examination time.

Therefore, it is important to determine the minimum number of images to average to obtain clear images of the retina. There have been studies that have analyzed the quality of an image as a function of the number of images averaged [3], [4]. The investigators compared the quality of images by subjective evaluations by the raters [3], [4]. While it may be relatively easy for a rater to determine the clearest image, it is not as easy to determine the quality of an image required to make a definitive diagnosis of some retinal diseases. In addition, there have not been any studies determining the minimum number of images to average to gain the clearest OCT image.

Thus, the purpose of this study was to determine the minimum number of SD-OCT images that have to be averaged to obtain a clear image. To eliminate subjective evaluations of the images, we used more objective methods by analyzing the standard deviations (SDs) and contrast-to-noise (CNRs) of the gray values of the different retinal layers. We shall show that averaging the B-scan images will reduce the SDs and increase the CNRs, but increasing the number of images to more than 20 did not lead to significant changes in the SDs and CNRs. We believe that our results can be used to maximize the image quality and minimize the exposure of the retina to infrared radiation.

## Methods

The study was approved by the Ethics Committee of Kagoshima University Hospital, and it was registered with the University Hospital Medical Network (UMIN)-clinical trials registry (CTR). The registration title was, “The effect of multiple B scans averaging in OCT imaging” and the registration number was UMIN000012286. All of the procedures conformed to the tenets of the Declaration of Helsinki. A written informed consent was obtained from all subjects.

### Subjects

A cross sectional prospective observational study was performed on 9 healthy volunteers. The eligibility criteria were; age 18-years or older, and eyes ophthalmoscopically normal. The exclusion criteria were; eyes with known ocular diseases such as glaucoma and diabetic retinopathy, subjects with known systemic diseases such as hypertension and diabetes, eyes with prior intraocular surgery or injections, and eyes in which the ocular fundus could not be observed due to media opacities. No eye was excluded due to poor OCT image quality caused by poor fixation.

Prior to the measurements, all eyes received a standard ocular examination which included slit-lamp examination of the anterior segment and funduscopic examination of the ocular fundus. The intraocular pressure (IOP) was measured with a pneumotonometer (CT-80, Topcon Corp, Tokyo, Japan). The best-corrected visual acuity (BCVA) was measured after determining refractive power of the eye with the Autorefractor Keratometer (RM8900, Topcon Corp).

### OCT Scanning Protocols

The Spectralis spectral-domain OCT (Heidelberg Spectralis-OCT, Spectralis; Heidelberg Engineering, Heidelberg, Germany) was used to obtain the images of the retina. All OCT scans were performed by experienced OCT operators. The baseline scanning protocol consisted of: scan extent  =  volume scan (15°×5°); scan sections  =  B-scans; 7 sections; and OCT automatic real time (ART) averaging. The ART was the number of frames averaged with a maximum of 100. For example, ART = 5 indicates that 5 SD-OCT images were averaged.

**Figure 1:**
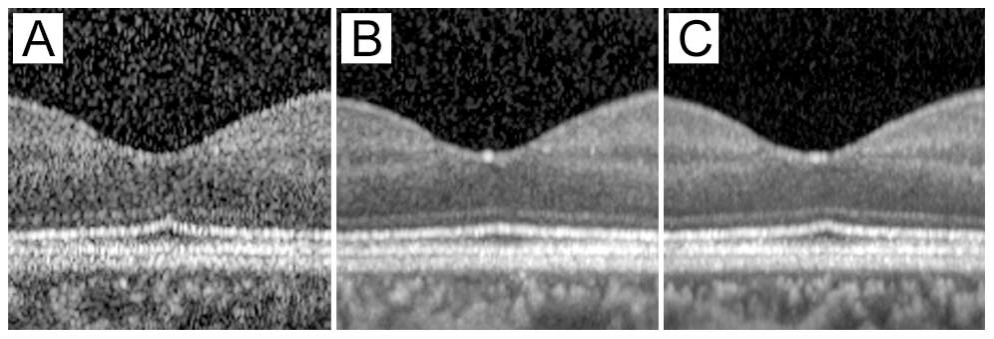
Representative SD-OCT images of fovea without (A) and with (B, C) automatic real time (ART) averaging. A: ART averaging  = 1; B: ART averaging  = 20; C: ART averaging  = 100. ART.

We set the baseline scanned image as the reference image and obtained the follow-up scans from the pre-set options selecting “Follow-up” from the OCT Acquisition Window to use the baseline scan as reference. The follow-up scans were made with the same settings as the baseline scans except the ART number was changed. The number of ART averaged in the follow-up scans was 1, 5, 20, 60, and 100. Baseline scans and all follow-up scans were obtained in less than 5 minutes (**Figure 1**).

### Analysis of OCT Images

**Figure 2:**
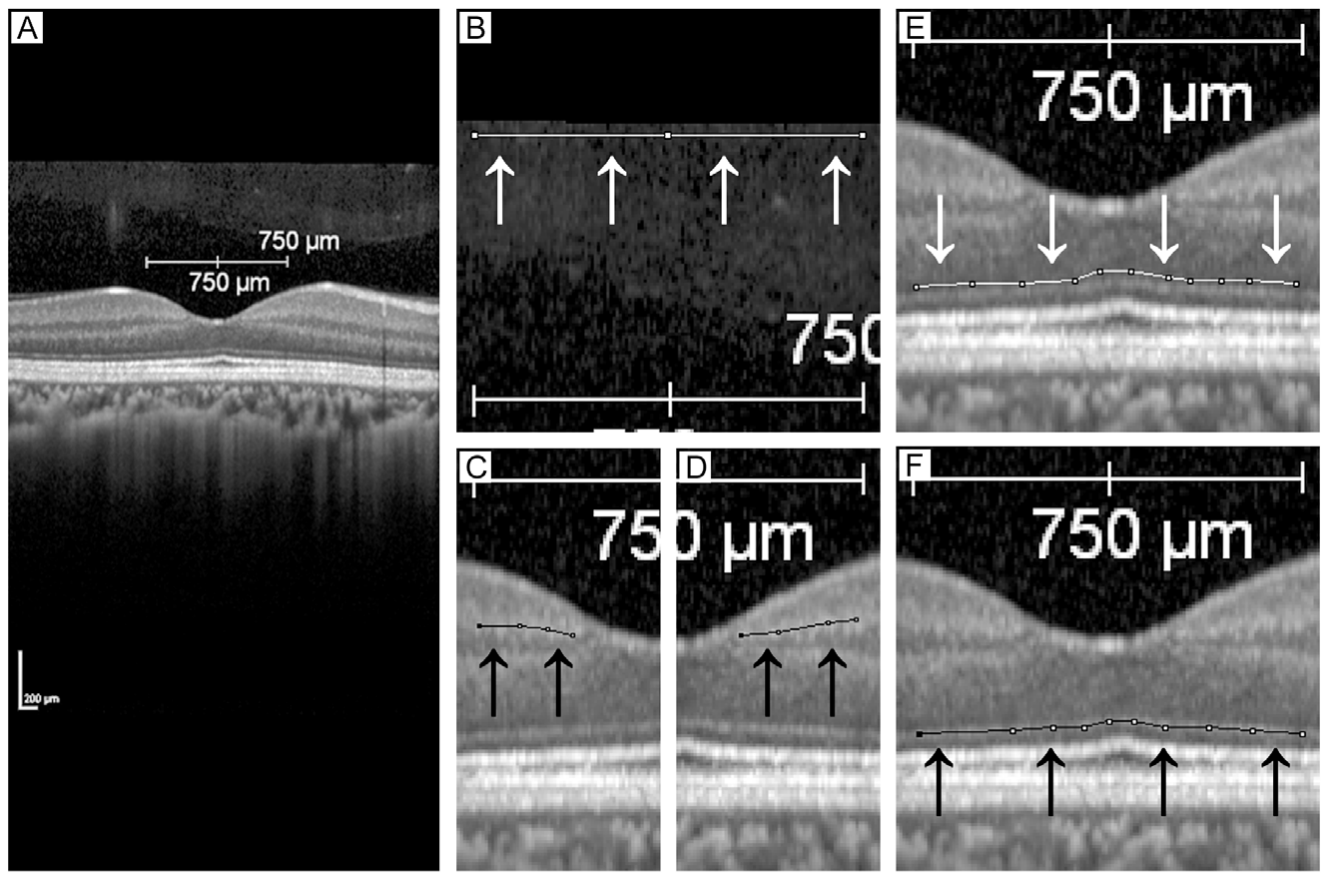
Region of interest (ROI) in each layer of a normal human retina. The region of interest is indicated by the arrows. A, original OCT image; B, vitreous was selected as ROI (white line); C. and D. IPL was selected as ROI (black lines); E. ONL was selected as ROI (white line); and F. ELM was selected as ROI (black line).

All averaged images were converted to 8-bit grayscale images by ImageJ version 1.47 (National Institutes of Health, Bethesda, MD; available at: http://imagej.nih.gov/ij/). ImageJ is a publically accessible free software to help analyzing images. A region of interest (ROI) is selected by the examiner as below and the ROI is analyzed as shown in Figure 2.

The brightness of each point was expressed in 256 levels from black  = 0 to white  = 255, and so the higher the brightness or gray value is, the brighter the image is. The mean brightness or mean gray values and the standard deviations (SDs) of the ROIs are then calculated. We selected a B-scan macular image through the fovea of the eyes, then set the ROIs manually on the vitreous body, inner plexiform layer (IPL), inner nuclear layer (INL), outer nuclear layer (ONL), external limiting membrane (ELM), photoreceptor inner segment/outer segment junction (IS/OS), cone outer segment tips (COST), and retinal pigment epithelium (RPE) (**Figure 2**). The reflectivity or the gray value of the ROI was expressed as a value between 0 and 255.

The reflectivity of each pixel on the ROI line was obtained and the mean gray value was calculated for the ROI. The SD was calculated by the following formula. 
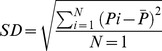
where *P* =  brightness of all pixels on the ROI line or the mean gray value), N =  number of pixels on ROI line, P1, P2, P3, …, Pi (i = 1, …, N)  =  the reflectivity of each pixel.

Each OCT image was measured twice by one grader (MS) for intra-rater repeatability, and they were also measured by two independent graders (MS, HT) for inter-rater reliability.

### Objective Assessment Parameters

We used two image parameters, the SD and the contrast-to-noise ratio (CNR) of the gray values of the ROIs, for the objective assessment of the clarity of the retinal layers. The SD of an image is the standard way of quantifying the amount of noise in an image and was calculated as described [2], [13]. The CNR assesses the contrast between two regions^3,6^ and is defined by the equation, 
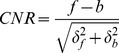
where *f* is the mean signal intensity (gray value) of the particular region in an image (foreground); *b* is the mean signal intensity of the surrounding region (background), and *δf* and *δb* are the standard deviations of the mean values of f and b. Higher CNR values would indicate greater contrast between adjacent retinal layers [3], [6].

### Phantom Eye Model

To eliminate the effect of ocular movements which occur during the recording of retinal images *in situ*, a “phantom eye” model was constructed according to a previous method with some modifications [14]. Thus, the “phantom eye” is the eye model made of silicone. Briefly, a silicone phantom retina was constructed of a stack of thin layered silicone plates [15]. Silicone (Sylgard 184, silicone elastomer, DOW Corning, Midland, MI, USA) is a hydrophobic, two-component product; a curing agent and silicone. The scattering properties of the silicone phantom plates are determined by the refractivity mismatches between the silicone, curing agent matrix, and suspended particles. To vary the scattering coefficients of the layers, we used different concentrations of titanium dioxide (TiO_2_) powder (Sigma Aldrich, St Louis, MO, USA). To obtain a homogeneous mixture, the suspended particles were mixed with the curing agent with a tissue homogenizer. Next, the mixture was placed in an ultrasonic bath for 10 minutes at 42 kHz to break apart the residual clusters. The mixture was mixed with silicone with careful stirring using a standard laboratory mixer, and the air bubbles were then removed with a vacuum pump. A small amount of the mixture was placed between two polytetrafluoroethylene (PTFE) plates separated by placing PTFE sheets of uniform thickness (50 µm) at the edges of the PTFE plates. Finally, the plates were cured at room temperature for 48 hours which resulted in a thin, single-layered phantom plate. Each phantom model was constructed with concentrations of titanium dioxide powder of 0%, 0.125%, 0.5%, and 2%, and the final phantom model was made by stacking four phantom plates of different concentrations of titanium dioxide.

The phantom eye model was kept stationary by a holder at the optimal position of the Spectralis OCT instrument. OCT images were obtained under the same condition as in human eyes, and the effect of averaging on image quality was evaluated by the SDs and CNRs as described for the *in situ* measurements.

### Statistical Analyses

All statistical analyses were performed with a commercial analytical package (SPSS Statistics 21 for Windows; SPSS Inc., IBM, Somers, NY). The intra-rater reliability was assessed by the intra-class correlation coefficients (ICC) using a one-way model and the inter-rater reliability was assessed by the ICC using a two-way model for absolute agreement. All of the the images including ART = 1, 5, 20, 100 of every subjects were analyzed when evaluating the ICC. Repeated measures analysis of variance (ANOVA) and Dunnett's post hoc tests were performed to compare the SDs and CNRs for each of the averaged images, i.e., the ART = 1, 5, 20, 60, and 100 images of the mean gray values. Repeated measures ANOVA and post hoc Tukey's honest significant difference (HSD) tests were performed to evaluate for the significance of differences in the SD and CNR values in the averaged OCT images. A value of *P* <0.05 was considered to be statistically significant.

## Results

### Demographics of Human Volunteers

Nine eyes of 9 volunteers were studied. Clear SD-OCT images of the retina were obtained from all subjects. Five subjects were women, and the mean ±SD age of the volunteers was 31.1±5.2 years with a range of 23 to 37 years. The mean refractive error (spherical equivalent) was −2.60±2.42 diopters (D) with a range from −7.50 D to +0.25 D.

### Intra-rater and Inter-rater Agreements

Both the intra-rater and inter-rater agreement for each layer was very high with a mean of ≥0.97 for all of the layers except the IPL and INL (**Table 1**).

**Table 1:** Intra- and inter-rater agreement of the reflectivity of SD-OCT images of a human eye.

	mean gray value		standard deviation	
ROI	intra-rater reliability†	inter-rater reliability‡	intra-rater reliability†	inter-rater reliability‡
Vit	0.995	0.992	0.991	0.987
IPL	0.893	0.890	0.888	0.907
INL	0.887	0.885	0.853	0.889
ONL	0.996	0.998	0.994	0.993
ELM	0.977	0.993	0.984	0.983
IS/OS	0.987	0.991	0.949	0.977
COST	0.982	0.985	0.953	0.971
RPE	0.992	0.996	0.951	0.971

ROI, region of interest; vit, vitreous body; IPL, inner plexiform layer; INL, inner nuclear layer; ONL, outer nuclear layer; ELM, external limiting membrane; IS/OS, photoreceptor inner segment/outer segment junction; COST, cone outer segment tips; RPE, retinal pigment epithelium.

† :intra-class correlation coefficients (ICC) using one-way model,

‡ ; ICC using a two-way model for absolute agreement. All *P* values are <0.001

### Effect of Averaging on gray value of ROI

#### Gray value of ROIs

**Figure 3:**
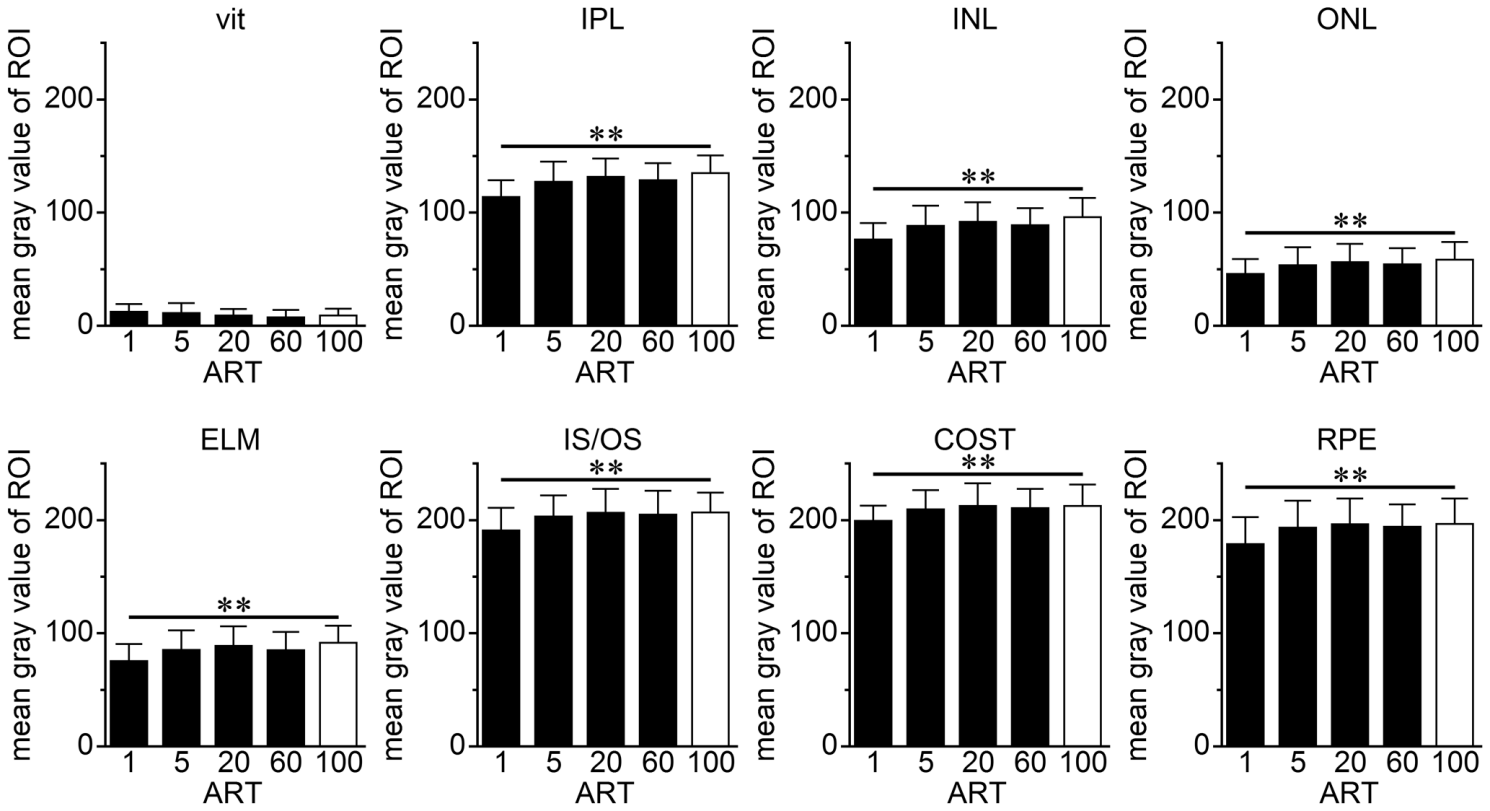
Gray values of SD-OCT reflectivity of each retinal layer of normal human eyes. The mean gray values of retina were significantly lower in the single scan image than in image averaged 100 times (mean gray value; **; *P*<0.01; post hoc Dunnett's multiple comparison test). ROI, region of interest; vit, vitreous body; IPL, inner plexiform layer; INL, inner nuclear layer; ONL, outer nuclear layer; ELM, external limiting membrane; IS/OS, photoreceptor inner segment/outer segment junction; COST, cone outer segment tip; RPE, retinal pigment epithelium. ART indicates automatic real time averaging.

The gray value of the vitreous did not change significantly after averaging up to 100 images (*P* = 0.083; repeated measures ANOVA). For the IPL, INL, ONL, ELM, IS/OS, COST, and RPE, the gray value of ART = 1 was significantly lower than that of ART = 100. By our definition using ImageJ software, the brightness of each point was expressed in 256 levels from black  = 0 to white  = 255. Thus, the higher gray values indicate a brighter image. These findings indicate that the ROI became brighter after 100 averages **(** **Figure 3**).

### Effect of Averaging on Noise Reduction

#### Standard deviation (SD) of reflectivity

**Figure 4:**
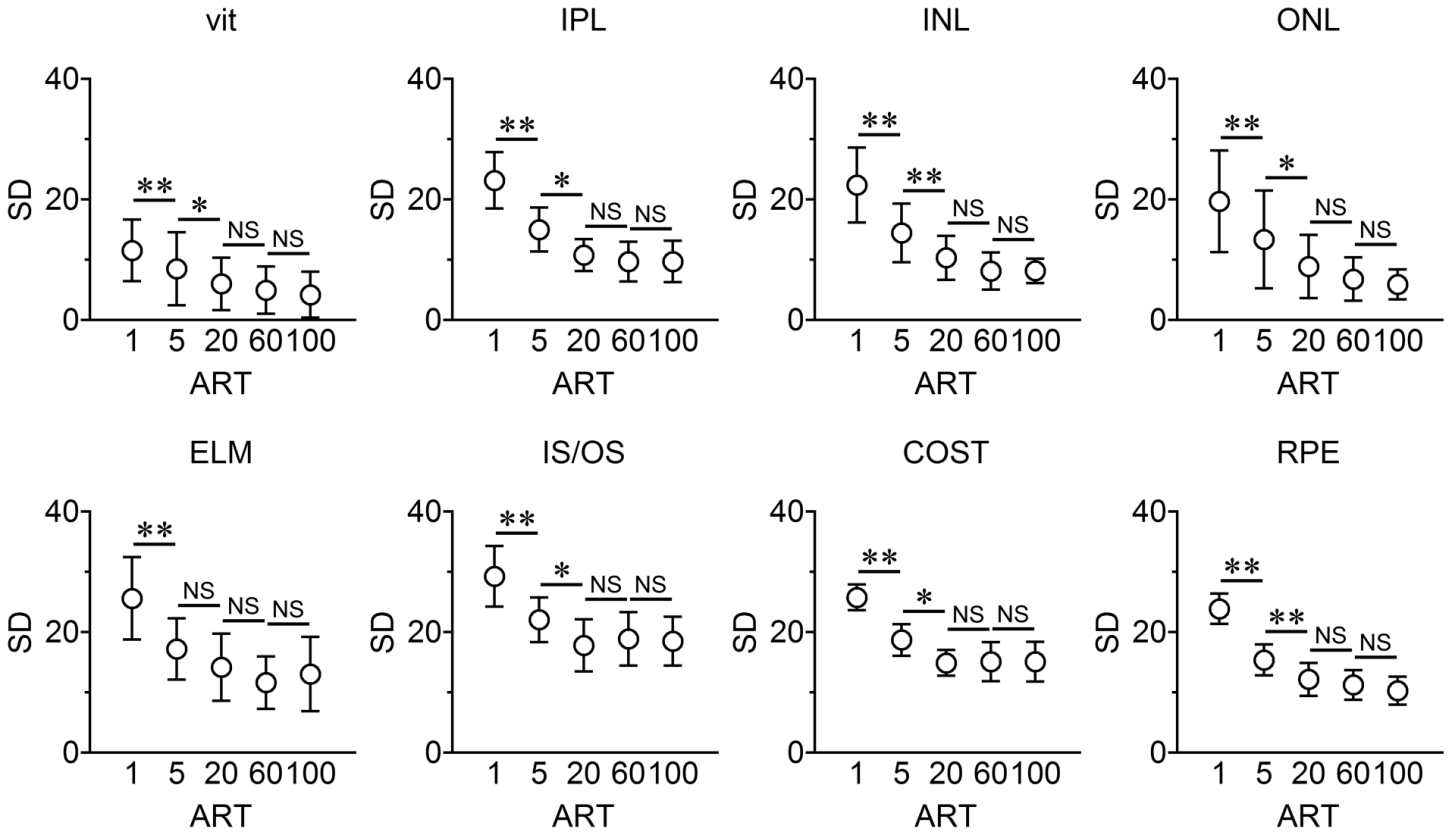
Standard deviations (SDs) after averaging in normal human eyes. The SD of the gray value of the OCT images decreases as the number of images averaged increases. ROI, region of interest; vit, vitreous body; IPL, inner plexiform layer; INL, inner nuclear layer; ONL, outer nuclear layer; ELM, external limiting membrane; IS/OS, photoreceptor inner segment/outer segment junction; COST, cone outer segment tip; RPE, retinal pigment epithelium. ART indicates automatic real time (ART) averaging. **; *P*<0.01, *:*P*<0.05, post hoc Tukey's HSD tests.

A high SD indicates higher speckle noise [5]. The SD decreased after each increase in the number of images averaged (*P*<0.01 for all: repeated measures ANOVA). The SD value for an image averaged 100 times (ART = 100) was significantly lower than that for either ART = 1 or ART = 5 (*P*<0.05; post hoc Tukey's HSD tests), but the difference in the SDs between ART = 20 and ART = 100 was not significant. Thus, ART = 20 is the optimal number of OCT images to average (**Figure 4** and **Table S1 in File S1**).

### Contrast-to-noise Ratio (CNR) of Reflectivity

The CNR was calculated from the mean gray values and the SDs of the different retinal layers. For these calculations, the gray values of the ELM, IS/OS, COST, and RPE layers were used as the foreground and that of the ONL as the background. For the IPL layer, the gray value of the IPL layer was used as the foreground and INL layer as the background.

The CNRs of the IPL/ONL, ELM/ONL, IS/OS/ONL, COST/ONL, and RPE/ONL increased significantly by increasing the ART numbers (all *P* values were <0.001 by repeated measures ANOVA).

**Figure 5:**
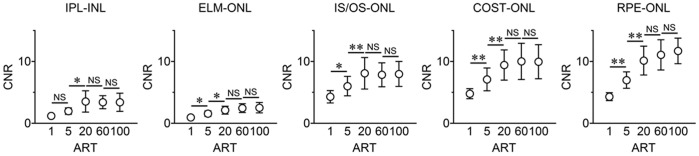
Contrast-to-noise ratios (CNR) of normal human eyes. Differences of CNR of OCT reflectivity after averaging between indicated layers was compared. The number of averaged number is expressed as the ART. The CNR increases with increasing the number of images averaged. The difference between ART = 5 and ART = 20 was significant. CNR, contrast to noise ratio; IPL, inner plexiform layer; INL, inner nuclear layer; ONL, outer nuclear layer; ELM, external limiting membrane; IS/OS, photoreceptor inner segment/outer segment junction; COST, cone outer segment tips; RPE, retinal pigment epithelium. ART indicates automatic real time averaging. *; *P*<0.05, **; *P*<0.01; post hoc Tukey's HSD tests.

For any of the foregrounds, the CNR of ART = 100 was significantly higher than that of ART = 1 or ART = 5 (*P*<0.01; post hoc Tukey's HSD tests), but not significantly higher than that of ART = 20 or ART = 60. The CNR of ART = 20 was significantly higher than that of ART = 5 (**Figure 5** and **Table S2 in File S1**).

### Phantom Eye Model

**Figure 6:**
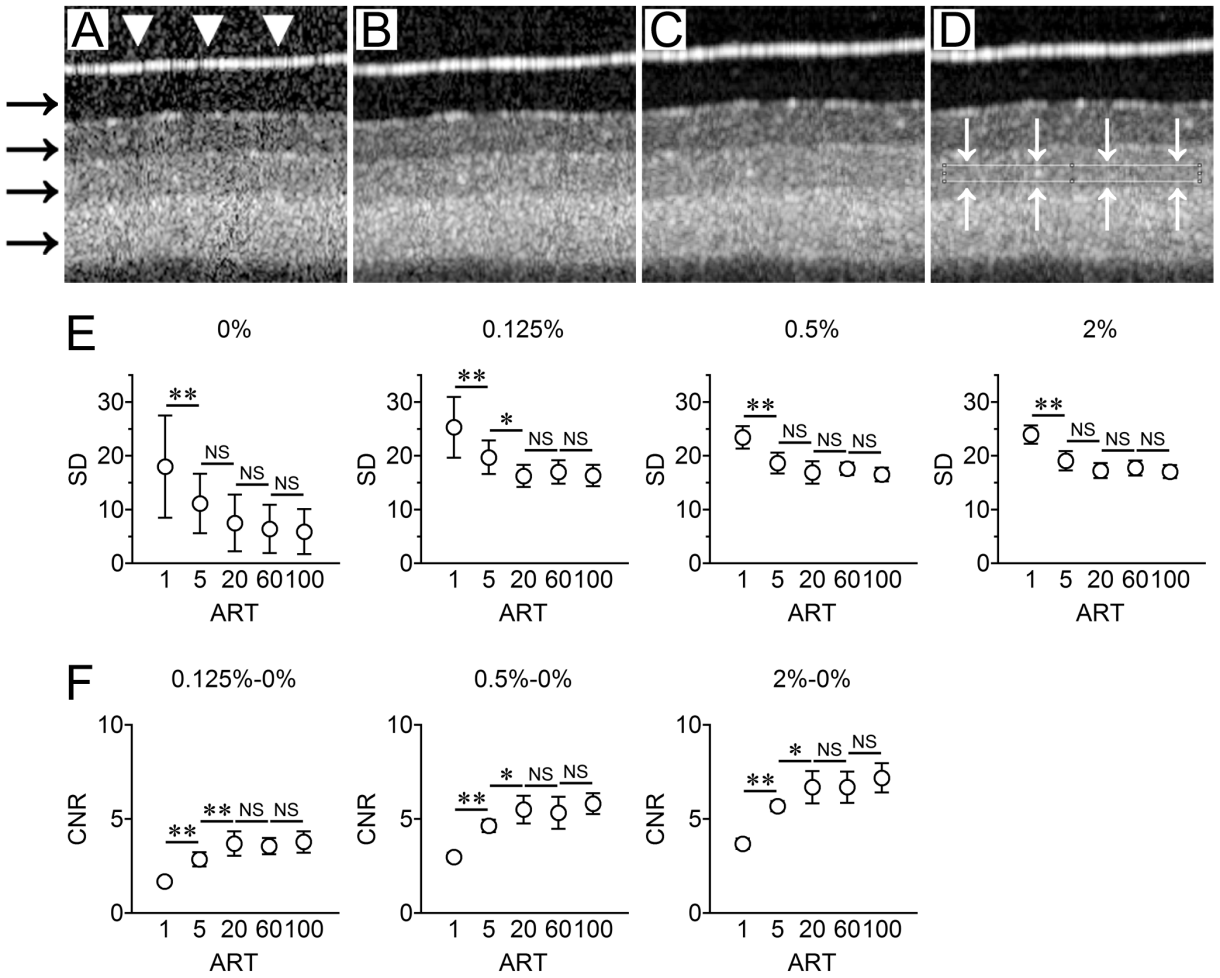
Results of phantom eye model. Representative SD-OCT images of phantom eye model (A–C). A, ART = 1; B, ART = 20; C, ART = 100. ART =  number of frames averaged. The phantom eye model is composed of four layers with different concentrations of titanium dioxide (TiO_2_) powder: 0%, 0.125%, 0.5%, and 2% (from the top to the bottom, black arrows). The upper surface appears as a hyperreflective line because of surface reflection (white arrowheads). The region of interest (ROI) in each layer of phantom eye model (D). D, TiO_2_ 0.5% layer was selected as ROI (white box). SD after averaging in phantom eye model (E). SD of gray reflectivity on OCT decreased according as increase of averaging number. **; *P*<0.01, *:*P*<0.05, post hoc Tukey's HSD tests. CNR of phantom eye model (F). CNR of each layer was significantly higher than in that of ART = 20 than that of ART = 1 or ART = 5 (*; *P*<0.05, **; *P*<0.01; post hoc Tukey's HSD tests.

The “Phantom eye model” was used to simulate the OCT images of human retina, and the four layers of the phantom eye were imaged by SD–OCT as in human eyes (**Figure 6** **, A-D**). The SD was significantly decreased after averaging from ART = 1 to ART = 5 (*P*<0.01: repeated measures ANOVA, **Figure 6** **, E**). The SD of images with 100 averages was significantly lower than either ART = 1 (*P*<0.05; post hoc Tukey's HSD tests, **Table S3 in File S1**), but not than that of ART = 20 or ART = 60.

For the CNR, the layer with 0% TiO_2_ was used as a reference and the CNR was significantly increased after averaging from ART = 1 to ART = 5 and from ART = 5 to ART = 20 (*P*<0.05: repeated measures ANOVA, **Figure 6** **, F**). The CNR of images averaged 100 times was significantly lower than either of ART = 1 or ART = 5 (*P*<0.01; post hoc Tukey's HSD tests, **Table S4 in File S1**), but not so than ART = 20 or ART = 60.

## Discussion

Our findings showed that the speckle noise in SD-OCT images can be reduced by increasing the number of B-scan images averaged. Of importance was that these finding were made by objective methods, viz., the SDs and CNRs of the gray values of each retinal layer. Similar findings were found in human eyes and a phantom eye model. To the best of our knowledge, this is the first study that used objective methods to show that increasing the number of SD-OCT images averaged decreased the speckle noise in the SD-OCT images. Our method consisted of measuring the SDs of the gray values of each retinal layer, and determining the CNR between adjacent retinal layers. Most importantly, our findings showed that increasing the number of images averaged to more than 20 images did not reduce the SD or increase the CNR significantly. Thus, averaging 20 SD-OCT B-scan images is the minimum number of images to obtain a clear retinal image.

It has been known for some time that the speckle noise is reduced by averaging the images, and increasing the numbers averaged led to an improvement of the image quality in computed tomography and magnetic resonance images [5], [7], [8], [16], [17]. This technique of averaging has also been used for OCT images of the retina, which were clearly shown by Pappuru et al [3] and Sakamoto et al [4]. In their studies, the image quality improved as the number of B-scan images averaged up to 16 or 20 images. The image quality was based on subjective evaluations by the raters and was greatly affected by the raters' experience and biases.

However, it has not been determined whether there was a limit in the number of images that should be averaged to improve the image quality. Because earlier studies used subjective methods to determine whether the image quality has improved, the results are questionable. Thus, we have used a more objective method of calculating the SDs of the gray values in the ROIs of the different retinal layers. The SD of the gray values is a standard way of quantifying the amount of noise in images [13]. A decrease in the SD means the image has less speckle noise which would then increase its clarity. We found that the SD decreased significantly in almost all of the retinal layers when the number of averaged images increased from 5 to 20. However, there was no significant decrease in the SD by increasing the number of images averaged by more than 20 (**Figure 4**). In addition, the SDs of the images averaged 5 times or fewer were significantly different (**Table S1 in File S1**).

We also calculated the CNR between adjacent retinal layers in which an increase in the CNR is an increase in the contrast between the adjacent layers [6]. The increase in contrast would indicate a clearer image. Our findings showed that the CNR increased as the number of images averaged increased, and significant differences were found between averaging 1 and averaging 5 and also between averaging 5 to averaging 20. However, increasing the number of images averaged beyond 20 did not lead to a significant improvement of the CNR (**Table S4 in File S1**).

Thus, our findings showed that the SDs decreased and the CNR increased as the number of images was increased. However, and most importantly, the SDs did not decrease significantly and the CNR did not increase significantly when the number of images averaged increased from 20 to 100. We can conclude that averaging 20 images will improve the image quality significantly, and averaging any more images will not improve the image quality significantly but only increase the testing time. Thus, 20 is the minimum number of images to average.

To evaluate the contrast of a specific retinal area, we selected the ONL as the reference background. We selected this layer because the OCT image quality of the ONL had the highest intra- and inter-rater agreement. In addition, its reflectivity had the lowest variance, i.e., lowest standard deviation. These characteristics should make it more suitable for its use as the reference background. Thus, we compared the CNR by averaging the differences between 100 summation and the others. The results indicated that there was a significant difference between ART = 100 and ART = 5, but not ART = 100 and 20 or larger (**Table S2 in File S1**).

There are several strengths of the present study. In the earlier studies, the quality of the images was determined in human eyes with various diseases. Because the OCT image quality is strongly influenced by various factors, such as the opacity of lens and/or vitreous body [18], these earlier studies did not evaluate only the averaging numbers. In our study, we studied eyes of healthy volunteer with no diseases. In addition, these eyes were relatively uniform, and we could exclude factors other than the averaging numbers as affecting the image quality.

Another important issue was the effect of eye movements on the image quality. In averaging, the effect of eye movement artifacts cannot be completely eliminated [4]. The results from the phantom eye model showed that the CNR improved with an increase in the number of images averaged, but it reached a plateau with more than 20 images averaged. Similarly, the value of SD decreased until 20 averages and reached a plateau. These patterns were similar to the results on the human eyes. In general, the longer time to record the multiple images, the greater chance of ocular movements occurring. Thus, we had hypothesized that eye movements would prevent an improvement of the OCT image quality when more than 20 images are averaged. However, the results from the study of phantom eye model showed that the improvement of image quality occurred adding up to 20 images, but not after that, in agreement with the results of human eye. Because a phantom eye model does not have any movement, we concluded that ocular movements or micro-saccades are not necessarily an important factor that can affect image qualities at least with the current Spectralis instruments.

Another strength of this study was its prospective design in which we studied 10 healthy subjects with a minimal number of factors that might degrade the OCT image. Scans of each eye were performed within a limited time which minimized the possibility of changes caused by diurnal variations and fluctuations of the intraocular pressure.

The limitations of this study were that the images were acquired only from young healthy subjects with no ocular pathology, and the findings do not necessarily reflect those of patients seen in a routine outpatient setting. These issues especially the age should be remembered in interpreting the results. Second, the present method was only partly objective [3], [4]. Although inter-rater agreement was found to be sufficiently high, the method was not perfectly objective. For example, the ROI is selected by the rater subjectively which might cause an uncontrollable bias. Third, we only evaluated the scanning protocol of the Spectralis SD-OCT. It is not known whether our observations can be generalized for other SD-OCT instruments and other scanning protocols. Fourth, the present study analyzed only retinal images and the results are not necessarily applicable to other structures such as the choroid. These limitations should be remembered in interpreting and generalizing the present results.

In conclusion, our findings showed that averaging 20 SD-OCT images will reduce the SD and increase the CNR of the gray values of the different retinal layers. Averaging more than 20 images did not improve the image quality significantly. We conclude that these objective methods to evaluate the image quality are a good method to evaluate the quality of an OCT image. These findings should be valuable for patients and physicians for minimizing the treatment burden and maximizing the image quality.

## Supporting Information

File S1 **Table S1–S4.** Table S1. Difference of standard deviation (SD) of each layer of averaged image numbers (vs ART = 100) in normal human eye. Table S2. Difference of CNR of two layers in averaging image numbers (vs ART = 100) in human eye. Table S3. Difference of SD of each layer in averaging image numbers (vs ART = 100) in phantom eye model. Table S4. Difference of CNR of two layers in averaging image numbers (vs ART = 100) in phantom eye model.
(DOCX)
