# Supplementary material for: Objective Determination of Optimal Number of Spectral-Domain Optical Coherence Tomographic Images of Retina to Average
Source: PLoS One. 2014 Oct 22;9(10):e110550. doi: 10.1371/journal.pone.0110550 (PMC4206300; doi:10.1371/journal.pone.0110550)
Supplement: File S1 — Table S1–S4. Table S1. Difference of standard deviation (SD) of each layer of averaged image numbers (vs ART = 100) in normal human eye. Table S2. Difference of CNR of two layers in averaging image numbers (vs ART = 100) in human eye. Table S3. Difference of SD of each layer in averaging image numbers (vs ART = 100) in phantom eye model. Table S4. Difference of CNR of two layers in averaging image numbers (vs ART = 100) in phantom eye model. (DOCX) [file pone.0110550.s001.docx]

**Supporting Information File S1**

**Table S1. Difference of standard deviation (SD) of each layer of averaged image numbers (vs ART=100) in normal human eye.**

| ROI | ART pair | *P** |
| --- | --- | --- |
| vit | 1 vs 100 | <0.001 |
|  | 5 vs 100 | <0.001 |
|  | 20 vs 100 | 0.136 |
|  | 60 vs 100 | 0.847 |
| IPL | 1 vs 100 | <0.001 |
|  | 5 vs 100 | <0.001 |
|  | 20 vs 100 | 0.652 |
|  | 60 vs 100 | 1 |
| INL | 1 vs 100 | <0.001 |
|  | 5 vs 100 | <0.001 |
|  | 20 vs 100 | 0.652 |
|  | 60 vs 100 | 1 |
| ONL | 1 vs 100 | <0.001 |
|  | 5 vs 100 | <0.001 |
|  | 20 vs 100 | 0.252 |
|  | 60 vs 100 | 0.968 |
| ELM | 1 vs 100 | <0.001 |
|  | 5 vs 100 | 0.006 |
|  | 20 vs 100 | 0.846 |
|  | 60 vs 100 | 0.692 |
| IS/OS | 1 vs 100 | <0.001 |
|  | 5 vs 100 | 0.049 |
|  | 20 vs 100 | 0.981 |
|  | 60 vs 100 | 0.998 |
| COST | 1 vs 100 | <0.001 |
|  | 5 vs 100 | 0.023 |
|  | 20 vs 100 | 1 |
|  | 60 vs 100 | 1 |
| RPE | 1 vs 100 | <0.001 |
|  | 5 vs 100 | <0.001 |
|  | 20 vs 100 | 0.159 |
|  | 60 vs 100 | 0.746 |

ROI, region of interest; vit, vitreous body; IPL, inner plexiform layer; INL, inner nuclear layer; ONL, outer nuclear layer; ELM, external limiting membrane; IS/OS, photoreceptor inner segment/outer segment junction; COST, cone outer segment tips; RPE, retinal pigment epithelium. * Tukey's honest significant difference tests.

**Table S2. Difference of CNR of two layers in averaging image numbers (vs ART=100) in human eye.**

| ROI | ART pair | P* |
| --- | --- | --- |
| IPL-INL | 1 vs 100 | 0.002 |
|  | 5 vs 100 | 0.071 |
|  | 20 vs 100 | 0.999 |
|  | 60 vs 100 | 1 |
| ELM-ONL | 1 vs 100 | <0.001 |
|  | 5 vs 100 | <0.001 |
|  | 20 vs 100 | 0.252 |
|  | 60 vs 100 | 0.997 |
| IS/OS-ONL | 1 vs 100 | <0.001 |
|  | 5 vs 100 | 0.004 |
|  | 20 vs 100 | 1 |
|  | 60 vs 100 | 0.998 |
| COST-ONL | 1 vs 100 | <0.001 |
|  | 5 vs 100 | 0.001 |
|  | 20 vs 100 | 0.922 |
|  | 60 vs 100 | 1 |
| RPE-ONL | 1 vs 100 | <0.001 |
|  | 5 vs 100 | <0.001 |
|  | 20 vs 100 | 0.119 |
|  | 60 vs 100 | 0.845 |

ROI, region of interest; IPL, inner plexiform layer; INL, inner nuclear layer; ONL, outer nuclear layer; ELM, external limiting membrane; IS/OS, photoreceptor inner segment/outer segment junction; COST, cone outer segment tips; RPE, retinal pigment epithelium. * Tukey's honest significant difference tests.

**Table S3. Difference of SD of each layer in averaging image numbers (vs ART=100) in phantom eye model**.

| ROI | ART pair | P* |
| --- | --- | --- |
| 0% | 1 vs 100 | <0.001 |
|  | 5 vs 100 | 0.006 |
|  | 20 vs 100 | 0.76 |
|  | 60 vs 100 | 0.995 |
| 0.13% | 1 vs 100 | <0.001 |
|  | 5 vs 100 | 0.056 |
|  | 20 vs 100 | 1 |
|  | 60 vs 100 | 0.978 |
| 0.50% | 1 vs 100 | <0.001 |
|  | 5 vs 100 | 0.076 |
|  | 20 vs 100 | 0.988 |
|  | 60 vs 100 | 0.651 |
| 2% | 1 vs 100 | <0.001 |
|  | 5 vs 100 | 0.09 |
|  | 20 vs 100 | 0.999 |
|  | 60 vs 100 | 0.889 |

ROI; region of interest, * Tukey's honest significant difference tests. ART indicates the number of automatic real time averaging.

**Table S4. Difference of CNR of two layers in averaging image numbers (vs ART=100) in phantom eye model.**

| ROI | ART pair | P* |
| --- | --- | --- |
| 0.125%-0% | 1 vs 100 | <0.001 |
|  | 5 vs 100 | 0.006 |
|  | 20 vs 100 | 0.76 |
|  | 60 vs 100 | 0.995 |
| 0.5%-0% | 1 vs 100 | <0.001 |
|  | 5 vs 100 | 0.002 |
|  | 20 vs 100 | 0.779 |
|  | 60 vs 100 | 0.414 |
| 2%-0% | 1 vs 100 | <0.001 |
|  | 5 vs 100 | 0.001 |
|  | 20 vs 100 | 0.568 |
|  | 60 vs 100 | 0.565 |

ROI; region of interest, * Tukey's honest significant difference tests
